# MiRNA-4537 functions as a tumor suppressor in gastric cancer and increases the radiosensitivity of gastric cancer cells

**DOI:** 10.1080/21655979.2021.1982843

**Published:** 2021-10-20

**Authors:** Jia Liu, Sili Yan, Jun Hu, Dong Ding, Yang Liu, Xia Li, Hai Song Pan, Gengxin Liu, Bo Wu, Yu Liu

**Affiliations:** a Department of Radiology, Hubei Provincial Hospital of Traditional Chinese Medicine, Wuhan, Hubei, China; b Department of Ultrasound Imaging, Hubei Provincial Hospital of Traditional Chinese Medicine, Wuhan, Hubei, China

**Keywords:** miR-4537, ZNF587, GC, radiosensitivity

## Abstract

Radiotherapy is a common method to treat gastric cancer (GC). However, the clinical outcomes of GC radiotherapy face challenges, and the mechanisms of GC radioresistance remain unclear. Our study aimed to investigate the role and mechanism of miR-4537 in the radiation sensitivity of GC cells. Cell viability was determined by Cell Counting Kit-8. The proliferation of HGC27 and KATO III cells was measured using a colony formation assay. Flow cytometry was performed to examine the changes in cell apoptosis. Western blotting was conducted to detect the expression of zinc finger protein 587 (ZNF587) protein in HGC27 and KATO III cells. To confirm the relationship between miR-4537 and ZNF587, a luciferase reporter assay was performed. MiR-4537 was downregulated in GC tumors and cells and suppressed cell proliferation, while promoting cell apoptosis in GC. Importantly, we found that miR-4537 reduced the radioresistance of GC cells. In addition, we also confirmed that miR-4537 expression is negatively correlated with ZNF587 expression in GC tissues. MiR-4537 bound to ZNF587 and suppressed the expression level of ZNF587. Overexpression of ZNF587 partially counteracted the effects of miR-4537 on cell proliferation and apoptosis. In conclusion, in GC cells, miR-4537 inhibited the ability of cell proliferation, but on the contrary, it promoted the ability of cell apoptosis and improved radiosensitivity of the cells.

## Introduction

GC patients are one of the largest groups of cancer patients worldwide [1]. Each year, the number of GC patients accounts for more than half of the entire East Asian cancer population [2]. With the continuous progress of understanding of the pathogenesis of GC, the treatments of GC include not only surgical resection, but also chemotherapy and radiotherapy and other treatment methods [3]. Radiotherapy is one of the main treatment methods for tumors and exerts a significant role in various gastrointestinal tumors [4]. In addition, the progress of radiotherapy before and after GC has received more and more attention. However, radiation resistance seriously impedes radiation therapy [5]. Therefore, to improve the effectiveness of radiotherapy, we need to develop new radiosensitizers.

At present, some biomarkers are used for the diagnosis or prognosis of GC, such as microRNAs (miRNAs), which are small noncoding RNAs and related to the development of cancers and the improvement of cancer radiotherapy [6–9]. For example, miR-196b enhances the effect of radiation treatment on GC cells [10]. MiR-203 promotes radiosensitivity of GC cells via zinc finger E-box binding homeobox 1 [11]. MiR-190b confers radiosensitivity through negative regulation of B cell leukemia/lymphoma 2 in GC cells [12]. According to the results of non-coding RNA profiling by array (GSE93415), miR-4537 was differentially expressed in GC tissues, compared to that in adjacent healthy gastric mucosa. However, the role and mechanism of miR-4537 in radiosensitivity of GC have not been discussed yet. We verified the downregulation of miR-4537 in gastric cancer, and thus chose miR-4537 as the research object.

In this study, we aimed to explore the effects of miR-4537 on the radiosensitivity of GC cells as well as the target gene downstream miR-4537 in GC cells. We hypothesized that miR-4537 may improve the radiation response of GC cells. Our study may provide novel insight into the improvement of radiotherapy in GCs.

## Materials and methods

### Tissues samples

Twenty pairs of gastric cancer (GC) tumors and adjacent-nontumor tissues were collected from Hubei Provincial Hospital of Traditional Chinese Medicine, which were then stored at −80°C immediately until RNA was extracted. All patients included in the study had no history of chemotherapy or radiation prior to surgery and signed informed consent forms. This study was approved by the Research Ethics Committee of Hubei Provincial Hospital of Traditional Chinese Medicine.

### Cell cultures and transfection

GC cell lines (KATO III and HGC27) were obtained from Cobioer Biosciences Co. Ltd (Nanjing, China). GC Cells were incubated with 10% fetal bovine serum (Gibco, CA, USA) in RPMI 1640 medium and 1% penicillin-streptomycin in a humidified atmosphere with 5% CO_2_ at 37°C. MiR-4537 mimics (5ʹ-UGAGCCGAGCUGAGCUUAGCUG-3ʹ) and negative control (NC) mimics (5ʹ-GUUGCAGUGUUGAGCCCGAGCA-3ʹ) were purchased from GenePharma (Shanghai, China) and were trasfected into GC cells using lipofectamine 3000 at room temperature for 48 h.

### Bioinformatics analysis

According to the results of GSE93415 (https://www.ncbi.nlm.nih.gov/geo/query/acc.cgi?acc=GSE93415), miR-4537 showed the most significantly abnormal expression in GC tissues (adjacent *p* value = 1.79e-08) and was further confirmed as a research object. Potential targets of miR-4537 were predicted from miRDB (http://mirdb.org/cgi-bin/search.cgi) under binding scores >80. MiRDB was also used for predicting the binding sites between miR-4537 and zinc finger protein 587 (ZNF587). The mRNA expression levels in 408 stomach adenocarcinoma (STAD) tissues and 36 normal tissues were obtained from GEPIA (http://gepia2.cancer-pku.cn/#index).

### Reverse transcription-quantitative polymerase chain reaction (RT-qPCR)

TRIzol reagent (Invitrogen) was prepared for extracting total RNA from tissues or cells. Reverse transcription from RNA to cDNA was performed using a TaqMan microRNA Reverse Transcription Kit (Biosystems, USA). SYBR green (Applied Biosystems) was used for quantitative real-time polymerase chain reaction. The experiment was repeated three times. U6 small RNA and glyceraldehyde-3-phosphate dehydrogenase (GAPDH) act as internal controls for miR-4537 and its target mRNAs, respectively. The relative expression levels of miR-4537 and ZNF587 were calculated by the 2^−ΔΔCt^ method [13]. Primer sequences were listed as follows:

miR-4537, forward: 5ʹ-ACACTCCAGCTGGGTGAGCCGAGCTGAGCT-3ʹ,

reverse: 5ʹ-TGGTGTCGTGGAGTCG-3ʹ;

U6, forward: 5ʹ-ATACAGAGAAAGTTAGCACGG-3ʹ,

reverse:5ʹ-GGAATGCTTCAAAGAGTTGTG-3ʹ;

ZNF587, forward: 5ʹ-GTGATGTGATGCTAGAGAACC-3ʹ,

reverse: 5ʹ-TTTGATCCACACCAACAACC-3ʹ;

GAPDH, forward: 5ʹ-TCAAGATCATCAGCAATGCC-3ʹ,

reverse: 5ʹ-CGATACCAAAGTTGTCATGGA-3ʹ.

### Fluorescence in situ hybridization (FISH)

The expression of miR-4537 was measured by a FISH kit (BIS-P0001, Guangzhou Bersin Biotechnology Co., Ltd., China). The cell slide was treated with Digoxigenin-labeled miR-4537 probe hybridization solution. Slide was hybridized at 42℃ for 16 h and washed with 2 × Saline Sodium Citrate Buffer, followed by immersion in 70% ethanol for 3 min and stained with 4-6-diamidino-2-phenylindole (DAPI) for 10 min. The slide was imaged using a fluorescence microscope (Leica, Germany).

### Immunohistochemical (IHC) staining

GC tissues were deparaffinized and rehydrated, and Target Retrieval Solution was used for antigen retrieval. Endogenous peroxidase activity was blocked by 0.3% hydrogen peroxide for 15 min. After blocking with 5% goat serum, the slides were incubated with rabbit polyclonal anti-ZNF587 (1:100, PA5-31408, Invitrogen) at 4°C overnight and then probed with HRP-conjugated goat anti-rabbit secondary antibody IgG (1:2000, ab205718, Abcam). Next, slides were incubated in 3, 3ʹ-diaminobenzidine and subsequently counterstained with Hematoxylin QS (Vector Laboratories).

### Immunofluorescence assays

Cells were fixed and permeated with 0.3% Triton X-100 at room temperature. Next, cells were blocked with bovine serum album in phosphate-buffered saline for 2 h at room temperature and incubated with a primary antibody against ZNF587 (1:100, PA5-31,408, Invitrogen) at 4°C overnight. After washing three times, cells were incubated with fluorescence-conjugated secondary antibody IgG. Cell nuclei were stained with DAPI. The staining was captured by a fluorescence microscope (Leica, Germany).

### Cell counting kit-8 (CCK-8) assay

GC cell lines KATO III and HGC27 were seeded in 96-well plates to evaluate cell proliferation. After being cultured for 24 h, 48 h and 72 h, cells were added with CCK-8 reagent (Dojindo, Japan) and incubated for 4 h. Cell proliferation was measured with CCK-8. A microplate reader (Bio-Rad, Hercules, CA, USA) was prepared for detecting the absorbance at a wavelength of 450 nm.

### Colony formation assay

The transfected KATO III and HGC27 cells were cultured for 48 hours, harvested, digested by trypsin, and resuspended into fresh RPMI‐1640 medium with 10% fetal bovine serum at a density of 500 cells per well. Next, 2 mL of cell suspension was plated into six‐well cell culture plates followed by 2‐week incubation under optimum conditions of 5% CO_2_ and 37°C. Cells were fixed with 4% paraformaldehyde and observed with 0.5% crystal violet (methanol solution) for 5 minutes. Microscope (Olympus, Tokyo, Japan) was used to observe the colony formation.

### Flow cytometry

GC cells (1 × 10^5^cells) were incubated in a six-well cell culture. After incubating for 48 h, KATO III and HGC27 cells were washed with 1× binding buffer and placed at room temperature in the darkness with staining of Annexin V/FITC (Annexin V-Alexa Fluor 488/PI Kit; Beijing 4A Biotech Co. Ltd. China) for 5 min. Finally, flow cytometer ACEA NovoCyte™ (Biosciences, China) was applied to analyze the apoptosis of stained cells.

### Luciferase activity assay

The wild-type 3ʹ untranslated region (3ʹ-UTR) fragment of ZNF587 (ZNF587-WT) or the mutated 3ʹ-UTR fragment of ZNF587 (ZNF587-MUT) was cloned into the firefly luciferase-expressing pGL3-Promoter vectors (Promega, Madison, WI, USA). KATO III and HGC27 cells were then transfected with 20 ng of indicated luciferase plasmid and 40 nM miR-4537 mimics or NC mimics with Lipofectamine 2000 (Invitrogen). Luciferase activity assay was analyzed with a luciferase assay kit (Promega) after the transfection for 48 h.

### Western blot

After protein extraction, sodium dodecyl sulfate polyacrylamide gel electrophoresis was used to separate proteins from the sample buffer. Next, the proteins were transferred to polyvinylidene difluoride membranes and blocked with 5% nonfat milk for 1 h at room temperature. The primary antibodies were purchased from Abcam company, including anti-ZNF587 (ab111697, 1:500) or anti-GAPDH (ab8245, 1:10,000). Membranes were incubated with the primary antibodies for a whole night at 4°C. Horseradish peroxidase-conjugated secondary antibodies (ab181658, 1:10,000) were used to incubate the membranes for 2 h at room temperature. In this study, GAPDH was regarded as a loading control. The protein blots were visualized using an ECL detection system (Pierce, Rockford, IL, USA).

### Statistical analysis

Spearman rank correlation method was used to detect the expression correlation between miR-4537 and ZNF587 in GC tissues. Student’s t-test was used for the difference comparison between two groups, and one-way analysis of variance was used for difference comparison between more than two groups. *P*< 0.05 indicated that the difference is statistically significant.

## Results

### MiR-4537 regulates the proliferation and apoptosis of GC cells

**Figure 1.**
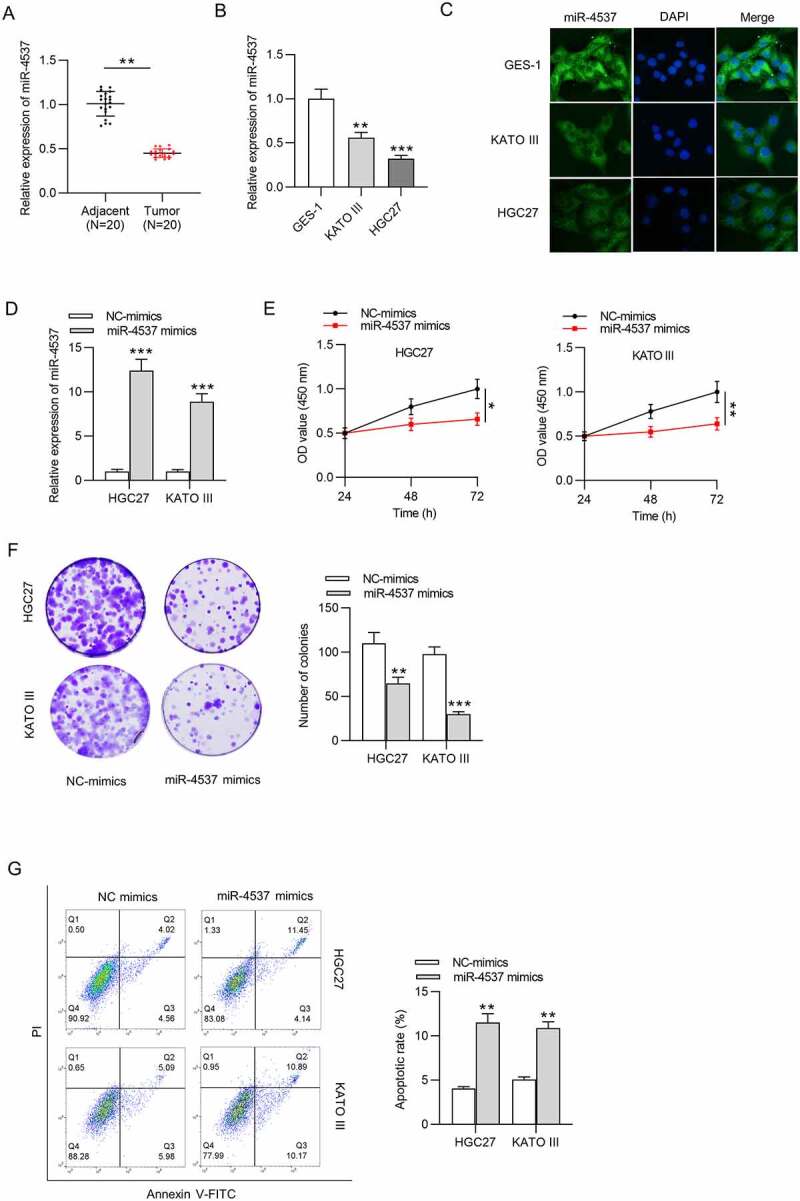
MiR-4537 regulates the proliferation and apoptosis of GC cells

We first detected the miR-4537 expression in GC and explored its influence on proliferation and apoptosis of GC cells. MiR-4537 expression level was decreased in 20 GC tissues, compared with the 20 adjacent nontumor tissues (Figure 1(a)). Similarly, miR-4537 expression levels in GC cell lines KATO III and HGC27 were significantly lower than that in control GES-1 cell line, as revealed by RT-qPCR and FISH assay (Figure 1(b-c)). Overexpression efficiency of miR-4537 was detected by RT-qPCR, which showed that miR-4537 mimics significantly upregulated the expression level of miR-4537, compared with the NC mimics group (Figure 1(d)). According to the results of CCK-8 assay, miR-4537 overexpression decreased the cell viability of HGC27 and KATO III, compared with the NC mimics group (Figure 1(e)). Colony formation assay results demonstrated that overexpression of miR-4537 repressed the colony formation abilities of HGC27 and KATO III cells (Figure 1(f)). Apoptosis rates of HGC27 and KATO III cells transfected with miR-4537 mimics were higher than those transfected with NC mimics (Figure 1(g)). These results indicated that overexpression of miR-4537 inhibited the proliferation and promoted the apoptosis of GC cells.

(a) RT-qPCR showed the expression level of miR-4537 in GC tissues. (b) The expression level of miR-4537 in GC cell lines was shown in RT-qPCR. (c) FISH assay was performed to reveal miR-4537 in GC cell lines. (d) Overexpression efficiency of miR-4537 was detected by RT-qPCR. (e) Viability of HGC27 and KATO III cells was determined using CCK-8 assay. (f) The colony-formation ability of GC cells transfected with miR-4537 mimics or NC mimics was detected by colony formation assay. (g) The apoptosis of GC cells transfected with miR-4537 mimics or control vector was measured by flow cytometry. *p< 0.05, ** *p* < 0.01, *** *p*< 0.001.

### MiR-4537 reduces the radioresistance of GC cells

**Figure 2.**
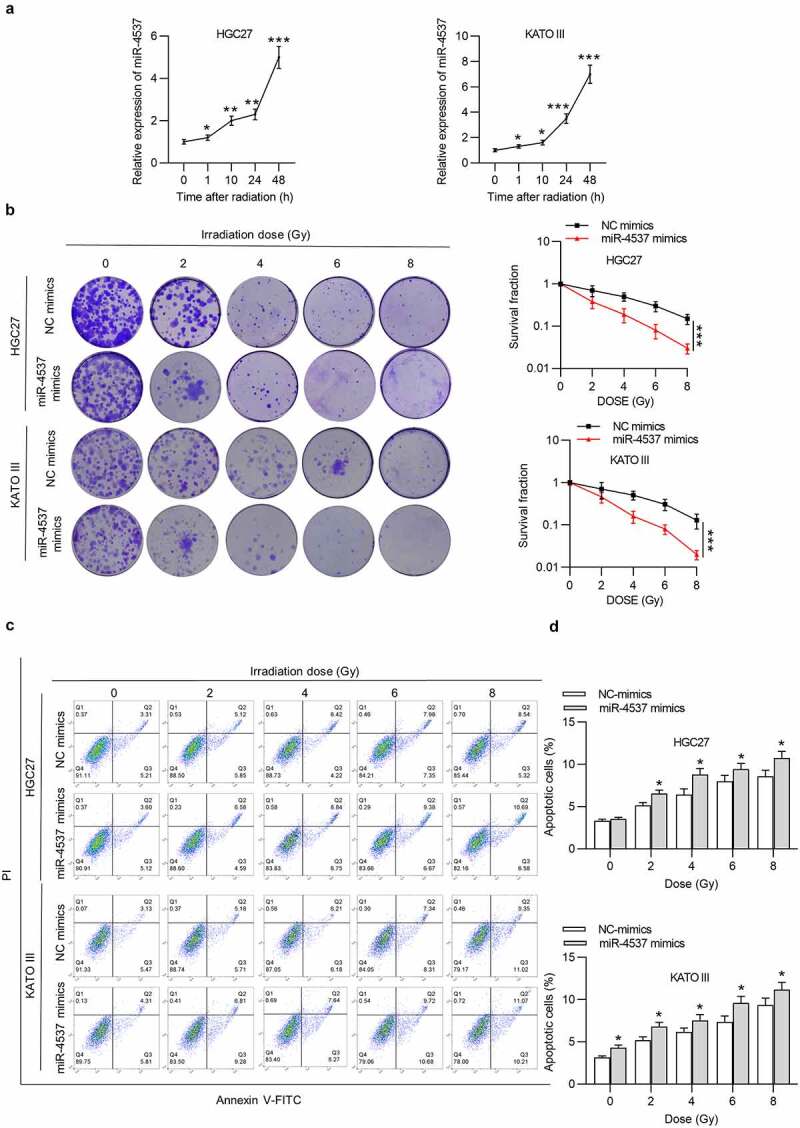
MiR-4537 reduces the radioresistance of GC cells

Effects of miR-4537 on the radioresistance of GC cells were detected. After radiation, the expression level of miR-4537 in GC cells was time-dependently increased in HGC27 and KATO III cells (Figure 2(a)). According to the colony formation assay, the number of colonies formed by the miR-4537 mimics was less than that in the control group in an irradiation dose-dependent manner. Based on the radiation dose–survival curve, the cells transfected with miR-4537 mimics were more sensitive to radiation treatment compared to cells transfected with NC mimics (Figure 2(b)). Flow cytometry results confirmed that irradiation dose-dependently increased apoptosis rate of GC cells, and miR-4537 mimics enhanced the promoting effects of irradiation on cell apoptosis (Figure 2(c-d)). Thus, overexpression of miR-4537 suppressed the radioresistance of GC cells.

(a) The expression of miR-4537 in GC cells was shown by RT-qPCR at different points of time after the irradiation. (b) The colony formation assay and the irradiation dose–survival curve were used to reveal the radiosensitivity of GC cells. (c) Cells were treated with irradiation dose, and cell apoptosis was detected by flow cytometry. (d) The apoptotic rate of each group was shown. *p< 0.05, ** *p* < 0.01, *** *p*< 0.001.

### ZNF587 is upregulated in GC tissues and cells

**Figure 3.**
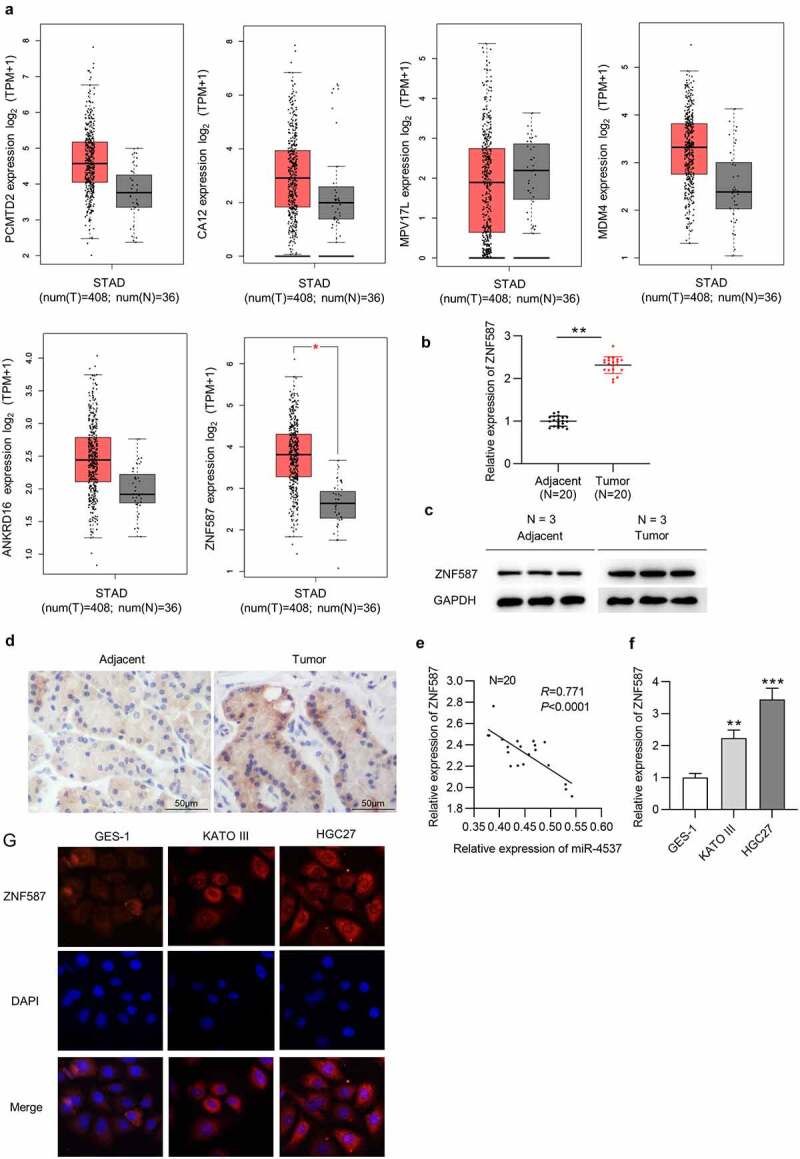
ZNF587 is upregulated in GC tissues and cells

Subsequently, we searched miRDB to explore the target genes of miR-4537. The mRNAs (PCMTD2, CA12, MPV17L, MDM4, ANKRD16, and ZNF587) with binding scores greater than 80 were identified (Table 1). GEPIA showed the expression levels of PCMTD2, CA12, MPV17L, MDM4, ANKRD16, and ZNF587 in 408 STAD tumors and 38 normal tissues. Among which, only ZNF587 had the significant difference in expression profile in tumor tissues and normal tissues (Figure 3(a)). Next, ZNF587 expression in GC tissues was detected. ZNF587 level in 20 GC tissues was higher than that in 20 adjacent nontumor tissues (Figure 3(b)). Results of western blotting and IHC staining revealed that ZNF587 protein level is higher in GC tissues than control adjacent tissues (Figure 3(c-d)). Spearman correlation analysis revealed that there was a negative correlation between miR-4537 and ZNF587 in 20 GC tissues (Figure 3(e)). ZNF587 was upregulated in HGC27 and KATO III cell lines, as revealed by the RT-qPCR and immunofluorescence staining assay (Figure 3(f-g)). Taken together, ZNF587 was upregulated in GC tissues and cells.

**Table 1.** Potential targets of miR-4537

Rank	Score	Gene Symbol	Gene Description
1	85	PCMTD2	protein-L-isoaspartate (D-aspartate) O-methyltransferase domain containing 2
2	82	CA12	carbonic anhydrase 12
3	82	MPV17L	MPV17 mitochondrial inner membrane protein like
4	82	MDM4	MDM4, p53 regulator
5	81	ANKRD16	ankyrin repeat domain 16
6	80	ZNF587	zinc finger protein 587

PCMTD2: protein-L-isoaspartate (D-aspartate) O-methyltransferase domain containing 2; CA12: carbonic anhydrase 12; MPV17L: MPV17 mitochondrial inner membrane protein like; MDM4: mouse double minute homolog 4; ANKRD16: ankyrin repeat domain 16; ZNF587: zinc finger protein 587. Note: targets were predicted from miRDB with scores more than 80.

(a) GEPIA revealed the expression levels of PCMTD2, CA12, MPV17L, MDM4, ANKRD16, and ZNF587 in STAD. (b-c) RT-qPCR and western blotting showed the relative ZNF587 expression in GC tissues. (d) IHC showed ZNF587 expression in adjacent nontumor tissues and GC tissues. (e) Spearman rank correlation was used to study the relationship between miR-4537 and ZNF587 levels in GC. (f) RT-qPCR showed the relative miR-4537 expression in cell lines. (g) Immunofluorescence staining of ZNF587 in control GES-1 cells and GC cells. ** *p* < 0.01, *** *p*< 0.001.

### MiR-4537 targets ZNF587 and negatively regulates ZNF587 expression

**Figure 4.**
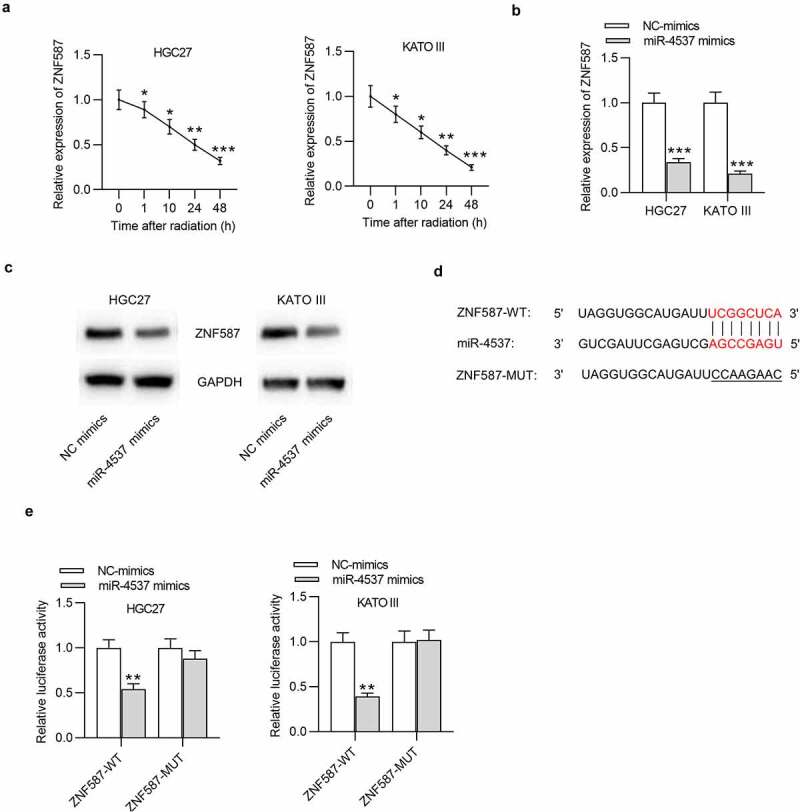
MiR-4537 targets ZNF587 and negatively regulates ZNF587 expression

Effect of miR-4537 on ZNF587 expression was detected. After the radiation, the expression level of ZNF587 was time-dependently decreased in both HGC27 and KATO III cells (Figure 4(a)). Overexpression of miR-4537 significantly suppressed the expression level of ZNF587, compared with the control NC mimics group (Figure 4(b)). Consistently, western blot also showed that the protein level of ZNF587 was reduced by miR-4537 mimics (Figure 4(c)). The binding site between miR-4537 and ZNF587 3ʹUTR was predicted by miRDB (Figure 4(d)). Luciferase reporter assay was conducted to further verify the binding between miR-4537 and ZNF587 3ʹUTR. We found that luciferase activities of HGC27 and KATO III cells transfected with ZNF587-WT were downregulated by miR-4537 mimics, but luciferase activities of cells transfected with ZNF587-MUT remained unchanged under the condition of miR-4537 mimics (Figure 4(e)). All these results indicated that miR-4537 targeted the 3ʹUTR of ZNF587 and negatively regulated ZNF587 expression.

(a) The expression of ZNF587 in GC cells was shown by RT-PCR at different points of time after radiation. (b) RT-PCR was used to detect the effect of miR-4537 mimics on the expression level of ZNF587. (c) Western blot was used to examine the effect of miR-4537 mimics on the protein level of ZNF587. (d) Binding sites between miR-4537 and ZNF587 3ʹUTR was shown. (e) The luciferase reporter assay was performed to verify the relationship between miR-4537 and ZNF587. *p< 0.05, ** *p* < 0.01, *** *p*< 0.001.

### MiR-4537 regulates the proliferation and apoptosis of GC cells by ZNF587

**Figure 5.**
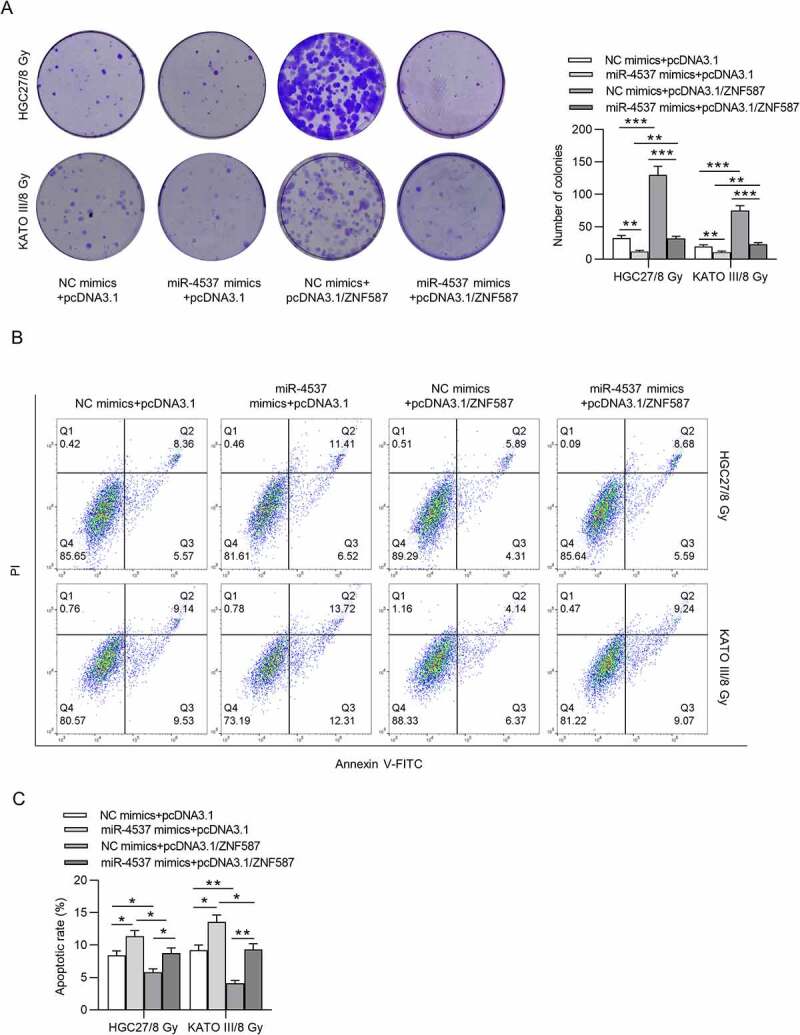
MiR-4537 regulates the proliferation and apoptosis of GC cells by ZNF587

In subsequent experiments, we overexpressed ZNF587 and conducted a series of functional rescue experiments to investigate whether ZNF587 has a rescue effect on miR-4537 in proliferation and apoptosis of GC cells. According to the results of colony formation assay, ZNF587 overexpression reversed the inhibitory effect of miR-4537 mimics on cell proliferation (Figure 5(a)). Flow cytometry revealed that ZNF587 overexpression rescued the increased apoptosis rate caused by the overexpression of miR-4537 (Figure 5(b-c)). Collectively, ZNF587 overexpression partially reversed the effect of miR-4537 on cell proliferation and apoptosis. Thus, miR-4537 regulated the proliferation and apoptosis of GC by ZNF587.

(a) Colony formation assay was used to detect the proliferation ability of transfected GC cells. (b-c) Flow cytometry was used to detect the apoptosis of transfected GC cells. *p< 0.05, ** *p* < 0.01, *** *p*< 0.001.

## Discussion

GC is a clinically common malignant tumor in the digestive tract [14]. With the improvement of medical technology, the treatment of GC has been continuously improved. However, the recurrence rate of GC after radiotherapy remains high. One of the causes for tumor recurrence is the presence of chemotherapy and radiation-resistant tumor cells [15,16].

It is well known that radiotherapy resistance greatly increases morbidity and mortality of GC patients, which becomes an important problem in the treatment of GC. Radiosensitivity is determined by the degree, repair ability, and other factors like oxygen consumption level, number of dividing cells and cell distribution at different stages of cell cycle. Radiation can cause DNA damage after directly affecting the sensitivity of tumor cells to radiotherapy [17]. How to sensitize GC cells to radiotherapy has always been the focus of research. In recent years, more and more miRNAs have been found to be associated with radioresistance [18–21], particularly in GC [22–24]. For example, miR-144-3p can increase the radiosensitivity of GC cells via zinc finger E-box binding homeobox 1 [25]. MiR-200 c is an effective radiosensitizer in GC cells [26]. MiR-195 and miR-503 are involved in cell resistance to radiation in GC [27]. However, up to now, few studies have explored the role of miR-4537 in radiotherapy sensitivity of GC. Here, we found that miR-4537 was downregulated in GC tissues and cells. Overexpression of miR-4537 suppressed cell proliferation, while promoting cell apoptosis. Importantly, radiation induced the upregulation of miR-4537 in a time-dependent manner. MiR-4537 mimics suppressed radioresistance of GC cells. Next, ZNF587 was confirmed as the target gene of miR-4537.

Zinc finger proteins (ZNFs) represent the largest transcription factor family, which are involved in diverse biological processes. Over the last few decades, increasing evidence has supported key roles of ZNFs in cancer occurrence and development [28]. For example, ZNF860 upregulation was an independent prognostic indicator in GC [29]. High expression of ZNF830 is associated with poor survival in lung and GC patients by mediating resistance to chemoradiotherapy [30]. Other ZNF members including ZNF281 [31], ZNF185 [32] and ZNF750 [33] serve as oncogenes or tumor suppressor genes in different cancers.

Since the significance of ZNF587 in GC development remains unclear, we further investigated it. In our study, ZNF587 was upregulated in GC tumors and cell lines. After the radiation, the expression level of ZNF587 was time-dependently decreased in GC cells. The downregulation of ZNF587 in GC cells after radiation exposure is caused by radiation-induced upregulation of miR-4537, which targets ZNF587 3ʹUTR, degrades ZNF587 mRNA, and suppresses its translation. In addition, ZNF587 expression was negatively correlated with miR-4537 expression in GC tumors. Notably, ZBF587 overexpression partially counteracted the effect of miR-4537 mimics on proliferation and apoptosis of GC cells.

There are also downsides in this study, such as no relevant experiments to determine the relationship between ZNF587 and GC cell radiosensitivity, and the lack of *in vivo* experiments to verify the results, which need exploration in the future. Moreover, the underlying mechanism of radiation-induced upregulation of miR-4537 remains unknown and deserves a further study.

## Conclusion

MiR-4537 regulated the proliferation and apoptosis of GC cells via ZNF587 and promoted the radiosensitivity of GC cells. Thus, our data indicated that miR-4537 was a potential target for regulating the development of GC cells and promoting the effect of radiotherapy on GC cells, which may help to improve the current therapeutic level of radiation.
